# Cyanobacterial Neurotoxin Beta-Methyl-Amino-l-Alanine Affects Dopaminergic Neurons in Optic Ganglia and Brain of *Daphnia magna*

**DOI:** 10.3390/toxins10120527

**Published:** 2018-12-08

**Authors:** Megan Brooke-Jones, Martina Gáliková, Heinrich Dircksen

**Affiliations:** Department of Zoology, Functional Morphology, Stockholm University, Svante Arrhenius väg 18B, S-10691 Stockholm, Sweden; megbrookejones27@gmail.com (M.B.-J.); martina.galikova@zoologi.su.se (M.G.)

**Keywords:** water flea, *Daphnia magna*, dopaminergic neurons, cyanobacterial toxin, BMAA, beta-methyl-amino-l-alanine, neurodegeneration

## Abstract

The non-proteinogenic amino acid beta-methyl-amino-l-alanine (BMAA) is a neurotoxin produced by cyanobacteria. BMAA accumulation in the brain of animals via biomagnification along the food web can contribute to the development of neurodegenerative diseases such as Amyotrophic lateral sclerosis/Parkinsonism dementia complex (ALS/PDC), the latter being associated with a loss of dopaminergic neurons. *Daphnia magna* is an important microcrustacean zooplankton species that plays a key role in aquatic food webs, and BMAA-producing cyanobacteria often form part of their diet. Here, we tested the effects of BMAA on putative neurodegeneration of newly identified specific dopaminergic neurons in the optic ganglia/brain complex of *D. magna* using quantitative tyrosine-hydroxylase immunohistochemistry and fluorescence cytometry. The dopaminergic system was analysed in fed and starved isogenic *D. magna* adults incubated under different BMAA concentrations over 4 days. Increased BMAA concentration showed significant decrease in the stainability of dopaminergic neurons of *D. magna*, with fed animals showing a more extreme loss. Furthermore, higher BMAA concentrations tended to increase offspring mortality during incubation. These results are indicative of ingested BMAA causing neurodegeneration of dopaminergic neurons in *D. magna* and adversely affecting reproduction. This may imply similar effects of BMAA on known human neurodegenerative diseases involving dopaminergic neurons.

## 1. Introduction

The non-proteinogenic amino acid β-methyl-amino-l-alanine (BMAA), was first identified from the seeds of *Cycas* trees on the island of Guam in the Pacific Ocean linked as a causative agent to the high incidence of amyotrophic lateral sclerosis/parkinsonism-dementia complex (ALS-PDC) among many of the indigenous Chamorro people of this island [1,2,3]. The cyanobacterium *Nostoc* living symbiotically with cycad trees was identified as the source for BMAA [2]. Another extensive source for BMAA was discovered in the skin of flying foxes, which devour large amounts of *Cycas* seeds and are regularly consumed as a food delicacy by the same indigenous people suffering from the neurological disease [4,5].

Several potential mechanisms by which environmental exposure to BMAA can lead to such neurological disorders have been proposed [6,7,8] and which are caused by neurodegeneration [9,10,11]. BMAA, however, is not only produced by many cyanobacteria but also by diatoms and dinoflagellates which occur almost ubiquitously in terrestrial, freshwater, brackish, and marine habitats [12,13,14,15]. BMAA has been detected in many freshwater lakes and brackish water bodies in Britain [16], in South African freshwater impoundments [17], and in urban waters bodies in the Netherlands [18]. BMAA even occurred in the desert sand of the Persian Gulf as a cause of neurological disorders in soldiers [19,20], in springs from a Gobi Desert oasis [21], as well as probably globally in freshwater and marine ecosystems [22,23]. Thus, there is mounting evidence for sporadic ALS or ALS/PDC being linked to environmental impact factors among which certainly cyanobacterial BMAA seems to be an important one as it is derived from knowledge about high incidences of ALS and dementia in Guam and the Kii peninsula of Japan [24,25,26,27]. These factors might even affect non-motor neurons in the disease pathology of ALS/PDC in susceptible individuals alongside the more familial genetic disease causes [28,29].

In particular, in freshwater environments in Sweden and in large swathes all around the Baltic Sea during summer months, algal blooms are a common phenomenon, and cyanobacteria and diatoms that also produce BMAA attain the highest concentrations [14,15,30,31]. Zooplankton organisms which naturally feed on different cyanobacteria usually contain higher levels of BMAA than the BMAA producers themselves [31]. In addition, recent studies have shown that fish and shellfish tissues from local markets in Sweden also contain higher concentrations of BMAA than the cyanobacteria [32,33]. Thus, these results clearly show that BMAA is biotransferred in aquatic ecosystems all around the world and accumulated in several aquatic organisms [34].

Bioaccumulation of BMAA has also been proven for *Daphnia magna,* one of the keystone organisms in the food web of freshwater ecosystems and likely the most common ecotoxicological test organism in the world [35,36,37,38]. *Daphnia* feeds on phytoplankton such as green algae but also on cyanobacteria, thereby being exposed to several cyanotoxins [36]. One of these cyanotoxins, BMAA, was later shown to adversely affect swimming behavior, reproductive output and survival especially upon exposure of the supposedly most sensitive neonate stages of *D. magna*, effects that were clearly dose-dependent [39]. However, nothing is known about the possible nature of these adverse effects on the physiology of *Daphnia*. In the present study, we set out to test the hypothesis of whether BMAA affects female fitness, locomotion, or causes neurological impairments. Because of the known links of BMAA and ALS/PDC syndromes as described above, we analyzed first whether dopaminergic neurons long known to be responsible for ALS/PDC syndromes in humans [40,41] may also be affected in *D. magna*.

The existence of dopamine as a transmitter in the *Daphnia* nervous system was evidenced long ago by the relatively unspecific Falck-Hillarp histofluorescence method [42] and later in *Daphnia* central nervous system (CNS) extracts [43]. More recently, dopamine and its rate-limiting enzyme tyrosine hydroxylase have been shown to have a role in modulating the motor behavior of *D. magna*, specifically their swimming movement [44]. Furthermore, other authors have proposed dopamine as a possible compound in some peripheral supposedly sensory cells and even found evidence for dopamine being involved in the expression of predator-induced defense in *Daphnia* [45]. We provide here the first description of identified dopamine neurons in *Daphnia* by the use of antibodies against tyrosine-hydroxylase, the rate limiting enzyme in dopamine biosynthesis. In fact, we found profound dose-dependent effects of BMAA on reduced dopamine activity in nearly all dopaminergic neurons of *D. magna* during short acute toxicity exposures.

## 2. Results

### 2.1. Identified Dopaminergic Neurons

**Figure 1:**
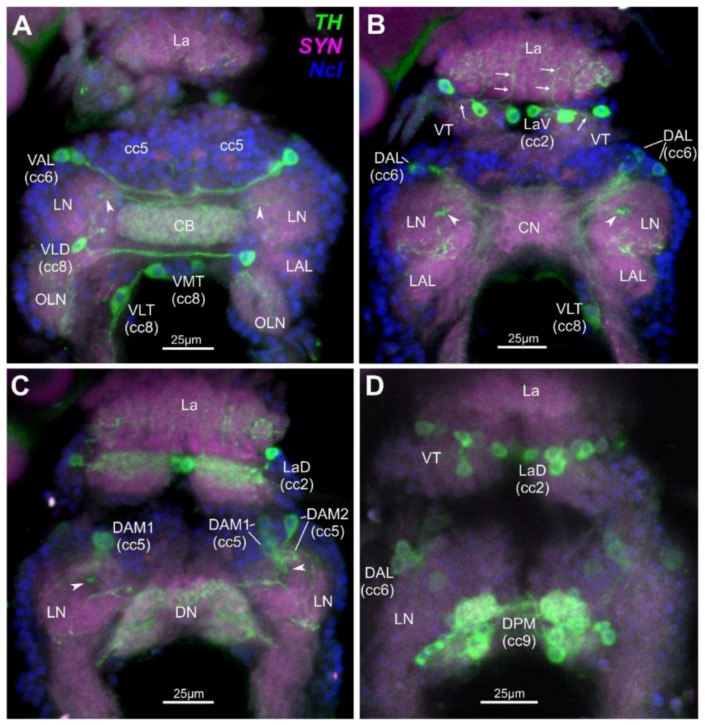
Dopaminergic neurons in the optic ganglia and central brain of *Daphnia magna* as revealed by tyrosine-hydroxylase (TH)-immunohistochemistry; ventral view; triple staining using fluoresceine-isothiocyanate-(FITC)-labelling for TH, green, AlexaFluor546-labelling for synapsin (SYN), magenta, 4′,6-Diamidine-2′-phenylindole (DAPI) nuclear stain (Ncl), blue. (**A**) Unipolar ventral anterior lateral (VAL), bipolar ventral lateral deutocerebral (VLD), bipolar ventral median tritocerebral (VMT) and bipolar ventral lateral tritocerebral (VLT) neurons; VAL partly innervate the anterior ventral central body (CB) and VLD innervate the ipsi- and contralateral anterior parts of the olfactory lobe neuropiles (OLN); 7 virtual 1 µm-sections combined. (**B**) Bipolar lamina neurons in ventral median to lateral positions (LaV), arrows indicate axons innervating single lamina cartridges and anterior areas of the visual tectum (VT); dorsal anterior lateral (DAL) neurons innervating the lateral neuropiles (LN); seven combined 1 µm-sections. (**C**) Dorsal anterior median (DAM1 and DAM2) innervating the LN, and DAM1 axons entering the funnel tract (arrowheads); eight combined 1 µm-sections. (**D**) Dorsal lamina (LaD) bipolar and dorsal anterior lateral (DAL) neurons, and the cluster of 8–10 dorsal posterior median (DPM) neurons, the latter innervating the dorsal neuropile (DN) and the posterior lateral neuropile (LN) (more details in **C**); three combined 1µm-sections. Arrowheads in (**A**,**C**) indicate axons from DAM1-neurons in the funnel tract innervating several layers of the CB. Abbreviations: cc, cell cluster; CB, central body; CN, central neuropile; DN, dorsal neuropile; La, lamina; LAL, lateral accessory lobe; LN, lateral neuropile; OLN, olfactory lobe neuropile; VT, visual tectum; nomenclature according to [46].

In total, a system of 25–27 paired dopaminergic neurons have been mapped in the optic ganglia/brain complex of *D. magna* (Figure 1). These comprise single neurons, some in groups of two or three and some in larger groups comprising up to 10 neurons. Neuron types have been attributed to five different cell clusters (cc) and are found to innervate several distinct neuroanatomical landmarks as defined earlier [46]. In the Lamina (La), 10 paired cells lie horizontally and make up the bipolar Lamina Ventral neurons (LaV) and bipolar Laminal Dorsal neurons (LaD) that lie in cc2 (Figure 1B–D). The axons of these cells innervate single cartridges in the La and anterior areas of the Visual Tectum (VT). In the anterior brain, two unipolar Ventral Anterior Lateral neurons (VAL) lie in cc6 which partly innervate the anterior ventral Central Body (CB) (Figure 1A), and three Dorsal Anterior Lateral neurons (DAL) innervate the Lateral Neuropiles (LN) (Figure 1B,D). Also, in the anterior brain, two types of Dorsal Anterior Median neurons (DAM1 and DAM2) which lie in cc5 innervate the LN. Axons from the DAM1 neurons project through the Funnel Tract (FT) from dorsal to ventral, finally innervating several layers of the CB (Figure 1A–C). Furthermore, in the deutocerebrum of the ventral brain, a single bipolar Ventral Lateral Deutocerebral (VLD) neuron lies posterior to the LN in cc8 and innervates the ipsi- and contralateral anterior parts of the Olfactory Lobe Neuropiles (OLNs) (Figure 1A). Also in cc8 and posterior to the VLD neuron in the tritocerebrum lies the single bipolar Ventral Median Tritocerebral neuron (VMT) and the single bipolar Ventral Lateral Tritocerebral neuron (VLT) (Figure 1A,B). Both cells have an ipsi- and contralateral projection with their respective axons into the tritocerebrum [46]. A cluster of 8–10 Dorsal Posterior Median neurons (DPM) lies in cc9, and the cells innervate the Dorsal Neuropile (DN) and Posterior LN (Figure 1C,D).

### 2.2. Effects of BMAA on Reproduction, Development and Behavior

**Figure 2:**
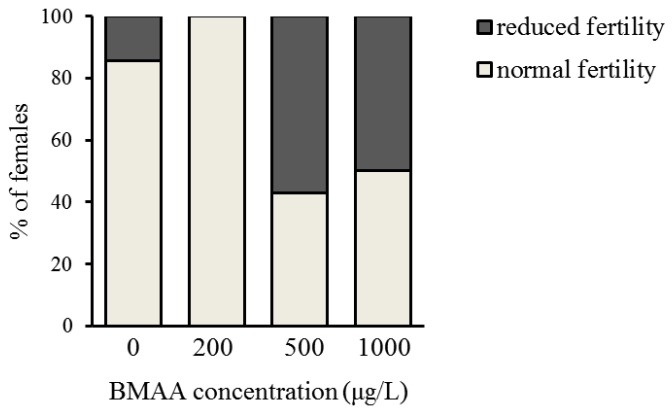
Exposure to beta-methyl-amino-l-alanine (BMAA) tends to decrease fertility of *D. magna* females. Fertility was defined as reduced if more than 50% of progeny was non-viable. Nonetheless, data do not reach statistical significance (pairwise comparisons with the 0 µg L^−1^ BMAA using Fischer’s exact test: *p* > 0.05 for each comparison). Sample size: 0 µg L^−1^ BMAA *n* = 7; 200 µg L^−1^ BMAA *n* = 6; 500 µg L^−1^ BMAA *n* = 7; 1000 µg L^−1^ BMAA *n* = 8.

**Figure 3:**
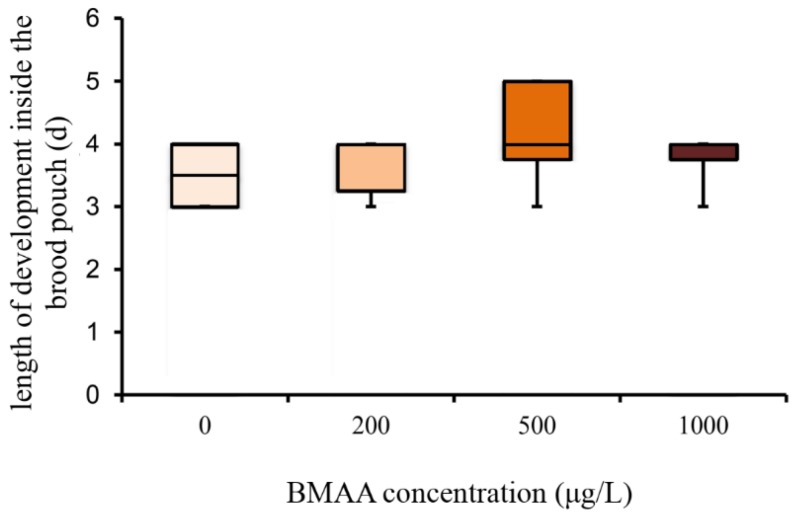
Beta-methyl-amino-l-alanine (BMAA) does not affect the length of development inside the brood chamber (one-way ANOVA, *p* > 0.05). Sample size: 0 µg L^−1^ BMAA *n* = 6; 200 µg L^−1^ BMAA *n* = 6; 500 µg L^−1^ BMAA *n* = 8; 1000 µg L^−1^ BMAA *n* = 4.

**Figure 4:**
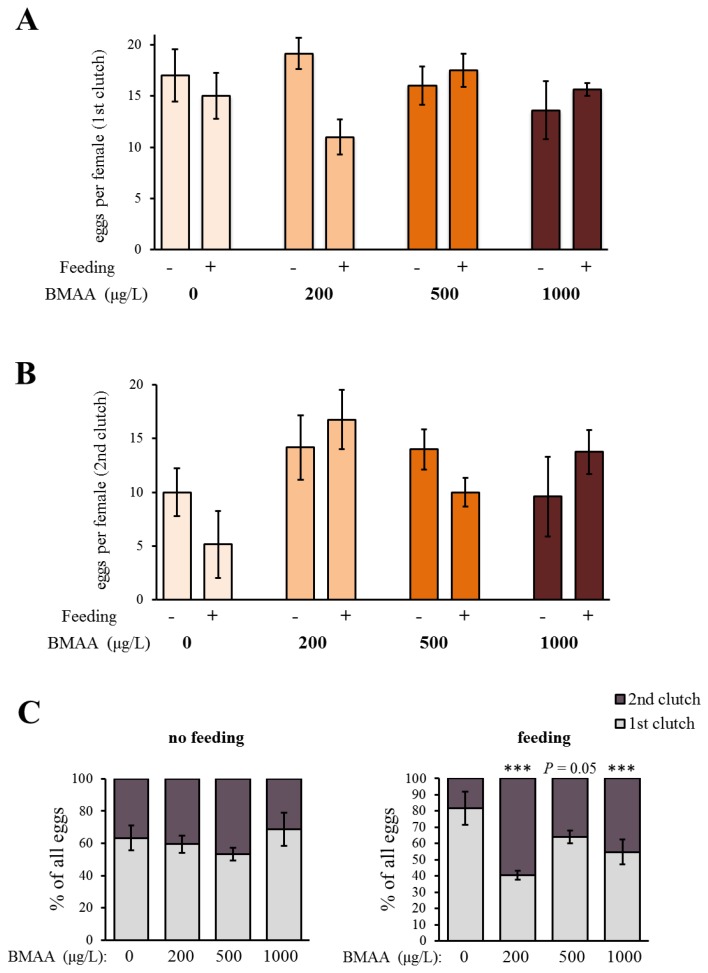
Beta-methyl-amino-l-alanine (BMAA) increases the number of eggs in the second clutch after the exposure to the toxin. (**A**) BMAA does not have any pronounced effect on the number of eggs in the first clutch after the exposure to the toxin (two-way ANOVA, feeding state: *p* > 0.05, BMAA: *p* > 0.05, feeding state × BMAA: *p* < 0.05). The toxin does not have any significant effect under the non-feeding stage (one-way ANOVA, *p* > 0.05), and under feeding conditions, the only detected difference was between the effects of the 200 and 500 µg L^−1^ BMAA (one-way ANOVA, *p* < 0.05; Tukey’s HSD, *p* < 0.05). Pairwise comparisons (two-tailed Student’s *t*-test, comparison to 0 µg L^−1^ BMAA) also did not reveal any significant decrease in egg production under exposure to BMAA (*p* > 0.05 for each comparison). (**B**) BMAA has an effect on the number of eggs in the second clutch after the exposure (two-way ANOVA, BMAA: *p* = 0.02, feeding: *p* > 0.05, BMAA × feeding: *p* > 0.05). BMAA does not affect the number of eggs in the second clutch under non-feeding condition (one-way ANOVA, *p* > 0.05), but significantly affects the number of eggs under feeding conditions (one-way ANOVA, *p* < 0.05) by increasing the number of eggs under exposure to 200 µg L^−1^ BMAA (Tukey’s HSD, *p* < 0.01). (**C**) BMAA does not affect the ratio between the eggs in the first and the second clutch after the exposure to the toxin in non-feeding animals (Fischer’s exact test, pairwise comparisons with the 0 µg L^−1^ BMAA, *p* > 0.05 for each comparison). BMAA increases the proportion of eggs in the second clutch in feeding animals (Fischer’s exact test, pairwise comparisons with the 0 µg L^−1^ BMAA: 200 µg L^−1^ BMAA: *p* < 0.001; 500 µg L^−1^ BMAA: *p* = 0.05; 1000 µg L^−1^ BMAA: *p* < 0.001). (**A**–**C**) Sample size for the non-feeding females: 0 µg L^−1^ BMAA *n* = 5; 200 µg L^−1^ BMAA *n* = 6; 500 µg L^−1^ BMAA *n* = 5; 1000 µg L^−1^ BMAA *n* = 5. Sample size for feeding females: 0 µg L^−1^ BMAA *n* = 6; 200 µg L^−1^ BMAA *n* = 8; 500 µg L^−1^ BMAA *n* = 8; 1000 µg L^−1^ BMAA *n* = 8. Error bars represent standard error of the mean (SEM).

In order to analyze the potential effect of the BMAA toxin on the fitness of *Daphnia*, we investigated the development and reproduction of females exposed to three different concentrations of BMAA (200, 500 and 1000 µg BMAA per L of medium). The toxin did not visibly affect the molting cycle of *D. magna*. Each adult animal successfully molted and shed a complete carapace between every release of young and before the next development of eggs in the brood chamber. However, the exposure to the high BMAA concentrations caused a tendency towards reduction of female fertility (Figure 2). Fertility was considered reduced if the female produced predominantly non-viable progeny. However, exposure to BMAA did not affect the length of development inside the brood chamber (Figure 3). Next, we analyzed whether BMAA affects female fecundity, i.e., the number of eggs inside the brood pouch. To analyze potential interactions between the toxin exposure and the nutritional status of the females, we tested females that were starved during the toxin exposure, as well as females that were fed ad libitum during the treatment. During the exposure time (4 days), females laid two clutches of eggs. We therefore analyzed the effect of feeding, BMAA treatment, and their interaction on both the first and the second clutch of eggs. Neither BMAA treatment nor feeding status affected the number of eggs in the first clutch (Figure 4A). Nevertheless, BMAA significantly affected the number of eggs in the second clutch in females that were fed during the treatment (Figure 4B), increasing fecundity of females exposed to the 200 µg L^−1^ BMAA. Interestingly, BMAA treatment increased the proportion of eggs in the second clutch upon all tested concentrations under feeding conditions (Figure 4C).

**Figure 5:**
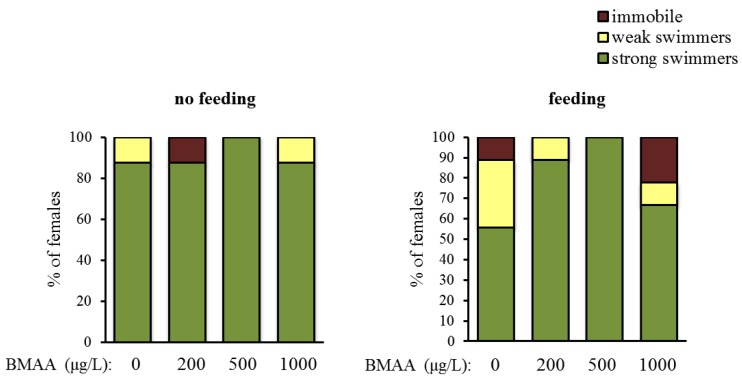
Beta-methyl-amino-l-alanine (BMAA) does not have a significant effect on the swimming ability, analyzed as differences in the % of females that are immobile, weakly and strongly swimming after 4 days of exposure to the toxin (χ^2^ test, *p* > 0.05 both under non-feeding and feeding conditions). Sample size for the non-feeding females: *n* = 8 for each concentration, sample size for the feeding females: *n* = 9 for each concentration.

Altogether, BMAA affects female reproduction. Next, we tested whether BMAA changes motility, which we analyzed as spontaneous swimming. Depending on their swimming capability, animals were categorized into three classes—immobile water fleas, weak swimmers and strong swimmers. Exposure to BMAA did not change the proportion of the categories, neither under starvation, nor under feeding conditions (Figure 5).

### 2.3. Effects of BMAA on Dopaminergic Neurons

**Figure 6:**
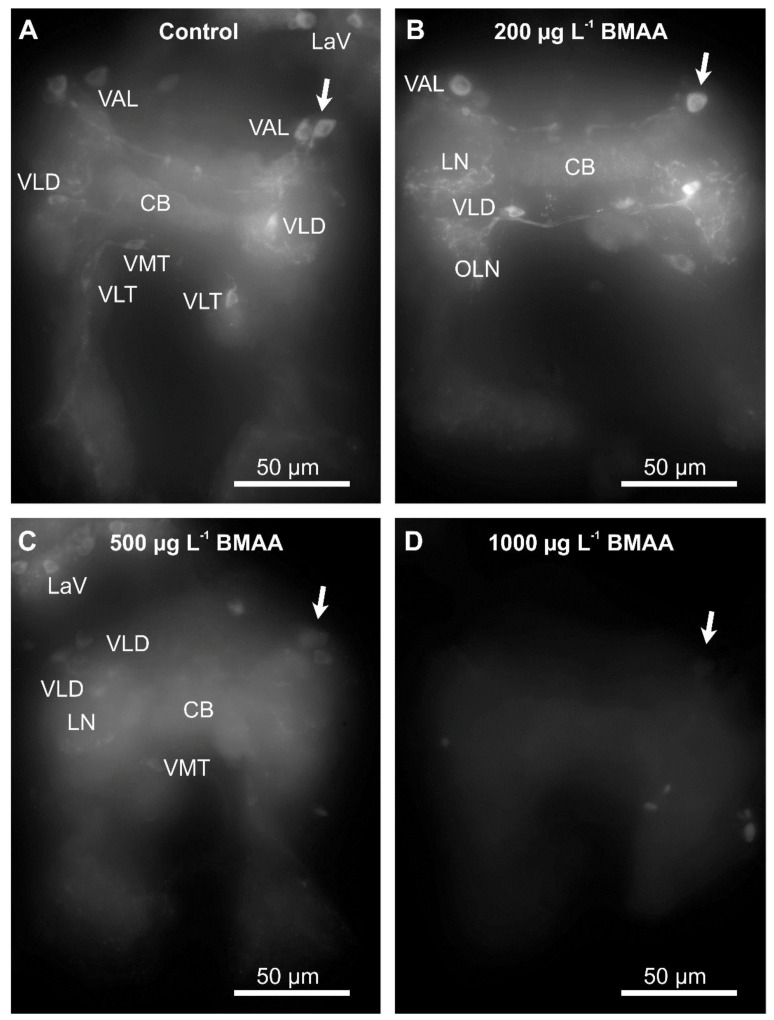
Beta-methyl-amino-l-alanine (BMAA) affects identified dopaminergic neurons in the brain cells of starved *D. magna* after 4 days of exposure to different concentrations of BMAA toxin. Identifiable neuron groups are labelled; arrows show identical unipolar ventral anterior lateral (VAL) neuron group as an example, cp. Figure 1A for abbreviations). (**A**) Control group, no BMAA added. (**B**) 200 µg L^−1^ BMAA exposure. (**C**) 500 µg L^−1^ BMAA exposure. (**D**) 1000 µg L^−1^ BMAA exposure. Note that the cell bodies of lamina neurons (e.g., LaV) and ventral neuron groups appear least affected by increased BMAA concentration, but neuropile fibers are also affected. Immunofluorescence demonstrated by the Zeiss Axioscope-2 microscope using the AxioCam HR camera with exposure time of 900 ms, 40× objective, 1.6× further magnification (via optovar) and 1 µm optical thickness in zeta-stacks.

**Figure 7:**
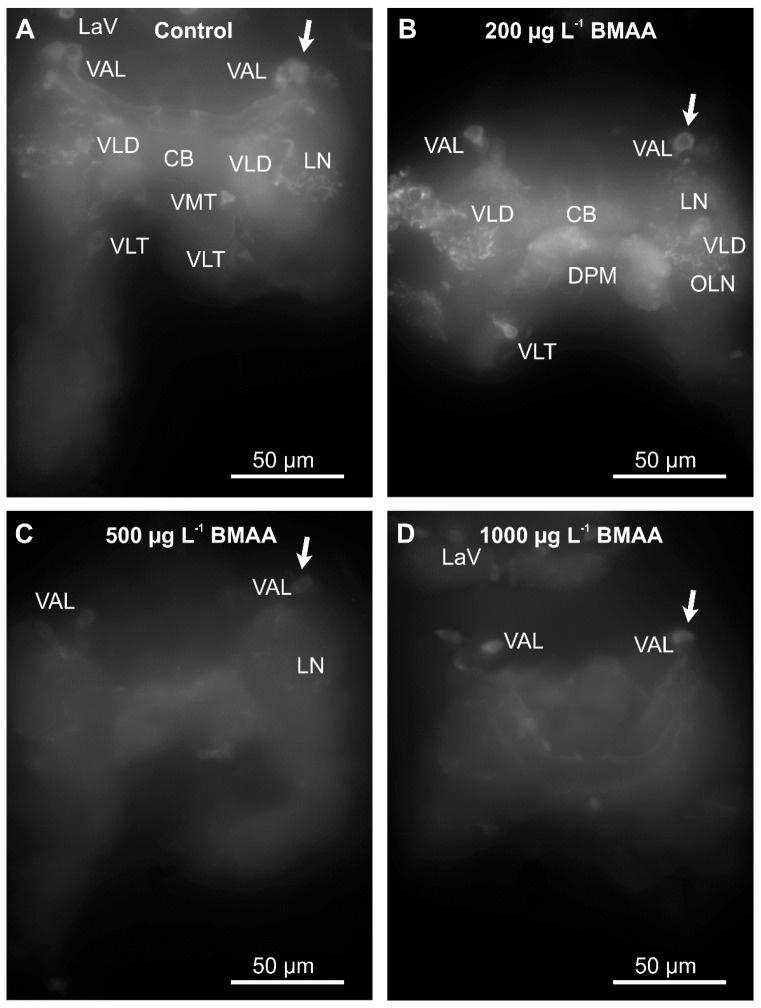
Beta-methyl-amino-l-alanine (BMAA) affects identified dopaminergic neurons in the brain cells of fed *D. magna* after 4 days of exposure to different concentrations of the BMAA toxin. Identifiable neuron groups are labelled; arrows show identical unipolar ventral anterior lateral (VAL) neuron group as an example, cp. Figure 1A for abbreviations. (**A**) Control group, no BMAA added. (**B**) 200 µg L^−1^ BMAA exposure. (**C**) 500 µg L^−1^ BMAA exposure. (**D**) 1000 µg L^−1^ BMAA exposure. Immunofluorescence as demonstrated by the Zeiss Axioscope-2 microscope and AxioCam HR camera using the same settings as in the legend to Figure 6.

**Figure 8:**
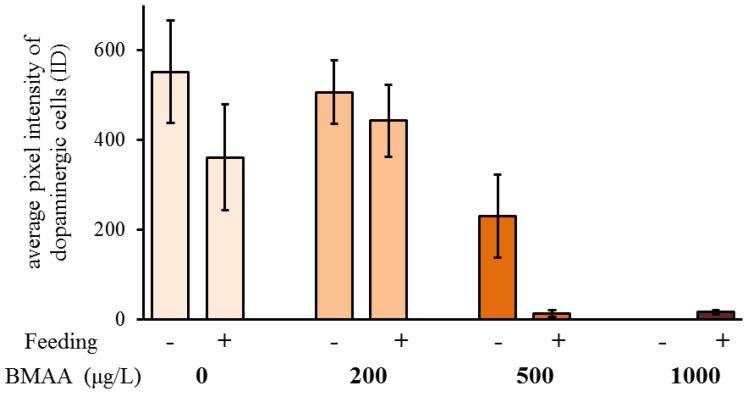
Beta-methyl-amino-l-alanine (BMAA) decreases immunofluorescence of the dopaminergic neurons, measured as integrated density (ID = fluorescent area of the dopaminergic neurons × their average pixel intensity). Both feeding state and BMAA treatment have a significant effect on the immunofluorescence of the dopaminergic neurons (two-way ANOVA, feeding state: *p* < 0.05, BMAA: *p* < 0.0001, feeding state × BMAA: *p* > 0.05). Sample size for the non-feeding females: 0 µg L^−1^ BMAA *n* = 16; 200 µg L^−1^ BMAA *n* = 10; 500 µg L^−1^ BMAA *n* = 13; 1000 µg L^−1^ BMAA *n* = 14. Sample size for the feeding females: 0 µg L^−1^ BMAA *n* = 14; 200 µg L^−1^ BMAA *n* = 11; 500 µg L^−1^ BMAA *n* = 17; 1000 µg L^−1^ BMAA *n* = 16. Error bars represent SEM.

Since we did not detect any impairment of mobility upon exposure to BMAA (Figure 5), we analyzed the potential effects of the toxin on the dopaminergic neurons directly, by analyzing their anatomy and dopamine levels by immunostainings, using an antibody against tyrosine-hydroxylase, the rate-limiting enzyme in dopamine biosynthesis. Exposure to the toxin led to a progressive decrease of immunofluorescence of the entirety of dopaminergic neurons in water fleas that were not fed during the exposure to the toxin (Figure 6), as well as in animals that were fed ad libitum during the treatment (Figure 7). Quantification of the immunofluorescence confirmed this negative effect of the BMAA (Figure 8). Increased concentrations of BMAA dramatically decreased the fluorescence intensity of dopaminergic neurons in *D. magna*. Since measurements of the entire sets of dopaminergic neurons were performed on flattened Zeta-stacks, the behavior of individual neuron types was no longer discernable. Nevertheless, at 500 µg L^−1^, a tendency was seen for dopaminergic lamina and some ventral neuron groups to apparently withstand BMAA treatment better than the dorsal groups (Figure 6 and Figure 7).

## 3. Discussion

A putative localization of dopamine in *D. magna* brain tissue has only been briefly described in the previous literature. The first evidence for dopaminergic neurons in *D. magna* brain and optic ganglia neurons was provided by the use of the Falck-Hillarp fluorescence technique [42]; it found reactivity in similar neuropiles and some cell groups as in the present study. The present study, however, has used a specific immunohistochemical technique to identify the entire dopaminergic system of *D. magna* for the first time, by use of an antibody against tyrosine-hydroxylase, the rate-limiting enzyme in the dopamine-synthesis pathway. The number and distribution pattern of dopaminergic neurons does not correlate to any other set of hitherto described neurons incl. neurosecretory neurons detected up to the present date in *D. magna.* Known neurosecretory and other systems include neurons producing crustacean hyperglycemic hormone [47], pigment dispersing hormone [48], allatostatin A-type, FMRFamide-related, tachykinin neuropeptides, and the neurotransmitters histamine [46] and serotonin [49].

The tendency towards lower fertility of *D. magna* in the acute toxicity test conducted in the present study correlates with increased mortality seen in other aquatic organisms when incubated in BMAA, like fish larvae *Danio rerio*, brine shrimps *Artemia salina* and the ciliate *Nassula sorex* [50]. These results also show substantial similarities with previous findings in *D. magna* neonates incubated for 48 h in BMAA [39]. This period is shorter compared with the 4-day incubation used in the present study, but their study found that immobility and mortality of *D. magna* neonates increased with increasing concentrations of BMAA. The present study used 4-day instead of 2-day exposures in order to cover the entire molting and reproductive cycle during adulthood (usually three days) and the possible influences. The present study did not find any significant effect of BMAA on the survival of the neonates, although a negative effect of BMAA on *D. magna* neonatal survival has been described previously [39]. Lürling et al. (2011) used the concentration from 100 µg L^−1^ BMAA to 1000 µg L^−1^ BMAA, but only at 1000 µg L^−1^ BMAA did the *D. magna* neonates show significantly increased mortality [39]. The differential outcome of this study vs. the work by Lürling et al. (2011) may have been caused by a different strategy of exposure of neonates to the toxin. Whereas Lürling et al. incubated *D. magna* neonates directly in BMAA [39], the present study looked at offspring neonatal survival produced from *D. magna* mothers incubated in BMAA. *D. magna* adults molt before each development of eggs in the brood chamber which is usually every 3–4 days [49]. Hence, 4 days of incubation in BMAA in the present study allowed time for each *D. magna* adult to molt at least once. The molting cycle of *D. magna* was not significantly affected by BMAA. Interestingly, in the females that have been fed during the toxin exposure, BMAA affected egg production. A mild exposure to the toxin increased the number of eggs in the second clutch. In addition, BMAA affected the ratio between the number of eggs in the first and second clutch. Under all tested concentrations, the number of eggs in the second clutch increased, in comparison to the first clutch. The analysis of the identified dopaminergic system of *D. magna* adults has clearly detected a significant decrease of dopaminergic neuron activity with increased exposure to BMAA. In both the fed and starved animals, a significant decrease of activity was seen in dopaminergic neurons of animals incubated in 500 µg L^−1^ and 1000 µg L^−1^ BMAA concentrations compared to the controls. The results showed that fed *D. magna* had very low dopaminergic neuron activity in both 500 µg L^−1^ and 1000 µg L^−1^ BMAA incubation whereas starved *D. magna* had very low dopaminergic activity only in the highest BMAA concentration of 1000 µg L^−1^. This suggests that feeding aids BMAA uptake because the fed *D. magna* are more affected by the toxins even at lower concentrations and hence may have taken up more BMAA. However, to obtain more distinctive results for individual dopaminergic neuron types or groups, their full reconstruction would be necessary to apply similar analysis methods.

Loss of activity of dopaminergic neurons in *D. magna* can be linked to neurodegenerative diseases in humans, as these diseases are associated with a loss of dopaminergic neurons [51,52]. Studying the effects of BMAA on dopaminergic neurons in *D. magna* in the present study has thus been an effective way to test the role of BMAA in neurodegenerative diseases, especially since this animal cannot avoid being affected in its aquatic environment. The BMAA hypothesis, first put forward from the investigations on Guam, states that BMAA could be a cause of neurodegeneration [53]. The results from this study on *D. magna* appear to support this BMAA hypothesis. In *D. magna*, dopaminergic neurons apparently have an integrated role in modulating motor behavior, specifically swimming movements [44]. Neurodegeneration of dopaminergic neurons could therefore explain the swimming deficits seen in *D. magna* neonates [39] during BMAA exposure. In humans, dopamine release in distinct brain neurons is important for motor control and decreased release of dopamine, especially when during neurodegenerative disease dopaminergic neurons are lost, can severely affect movement including reduced gait speed, shorter step length and prolonged standing phase [54,55]. The similarities of the role of dopamine in *D. magna* and humans, and the consequential behaviors that are observed when dopamine is decreased in both species demonstrates that *D. magna* is an adequate model organism to test toxicity effects leading to neurodegeneration closely similar to that known in humans. *D. magna* would even be capable of passing BMAA along the food chain from cyanobacteria (or from BMAA-containing green algae) via fish or shellfish predators to the human diet leading to BMAA biomagnifications as seen in the analyses of Swedish seafood [32,33].

In conclusion, the present study has proven *D. magna* to be an adequate model organism for toxicity testing of BMAA at the level of identified neurons. These results extend our knowledge of the crucial link between human neurodegenerative diseases and their being influenced by the environment, and they raise the ecotoxicological aspects of possible disease acquisition pathways. With the incidence of cyanobacterial blooms prone to increase worldwide due to global warming [56], it is now more important than ever that further investigations into the neurotoxic effects of BMAA are completed.

## 4. Materials and Methods

### 4.1. Experimental Animals

All animals used in the present study were cladoceran *Daphnia magna* (Strauss 1820), the test strain for environmental pollution, “Klon 5” from the State office for Nature, Environment and Customer Protection (North-Rhine Westphalia, Bonn, Germany) originating from the Federal Environmental Agency (Berlin, Germany). They have since been cultured in the laboratory at the Department of Zoology of Stockholm university (Sweden) in tap water in 1 L vials in a climate chamber with photoperiod set to a 16 h light: 8 h dark cycle and temperature at 20 ± 2 °C under ca. 1200 Lux artificial light. These animals were fed ad libitum the green algae *Scenedesmus subspicatus* (Chodat 1926) and *Chlorella vulgaris* (Beijerinck 1890) that were axenically reared under normal daylight in a defined culture medium (DIN 38412-9.1).

Prior to the experiments, a new isogenic female lineage of *D. magna* was set up. Six parthenogenetic females from one mother were incubated in 1 L glass vials, with one animal in each, containing 750 mL reconstituted artificial lake water medium (8 mg L^−1^ KCL, 192 mg L^−1^ NaHCO_3_, 245.95 mg L^−1^ MgSO_4_ × 6 H_2_O, 120 mg L^−1^ CaSO_4_, MilliQ-filtered H_2_O), as standardized by the American Society for Testing and Materials (ASTM) with 4 mL L^−1^ of added Scottish marine algae extract (Scotland) (at optical densities between 0.56–0.60 as measured photometrically at 400 nm wave length) [57]. The medium was exchanged every 3 to 4 days and the animals were fed ~8.5 × 10^7^ mL^−1^ *S. subspicatus* and ~9.6 × 10^7^ mL^−1^ *C. vulgaris* daily. The neonates of the first synchronized brood were isolated and separated into different vials; these were allowed to grow in the medium until they themselves produced a synchronised brood, the neonates of which were then isolated and separated again into 1 L vials. This third generation of parthenogenetic females (all of the same age ± 12 h) was grown for 10 days until adulthood to be used in the experiments.

### 4.2. BMAA Toxicity Test

Previous work had shown that at lower toxin concentrations, *D. magna* are better at taking up dissolved BMAA rather than BMAA in cyanobacteria [35]. Accordingly, the present study used only dissolved BMAA instead of cyanobacteria as a food source. For the toxicity test, 68 adult parthenogenetic females of *D. magna* were separated individually into 100 mL glass vials containing 50 mL of ASTM medium and 20 µL Scottish marine algae extract. The animals in vials 1 to 17 were kept as controls with no BMAA toxin added. Previous work had shown that after being exposed to 100 µg L^−1^ BMAA over 48 h, 50% of *D. magna* neonates became immobile (showing heartbeat but no swimming) [39]. Following these results, the present study incubated animals in concentrations of 200, 500 and 1000 µg L^−1^ BMAA for 4 days. An incubation time of 4 days was chosen in order to allow each animal to molt once to see if BMAA has effects on or during molting.

In addition, the effects of feeding on BMAA uptake were investigated. Therefore, half of the animals from each independent group were fed and half were starved (last fed 24 h prior to incubation start). Animals that were fed were given high food levels (5 × 10^5^ mL^−1^) of *S. subspicatus* and (5 × 10^5^ mL^−1^) *C. vulgaris* daily. The concentration of both *S. subspicatus* and *C. vulgaris* was calculated as follows: 4 µL of each alga was placed onto glass slides under 20 × 20 mm^2^ cover slips, to cover the whole cover slip. Ten photographs were taken at random with the 20× objective and at 1.0× magnification of each alga with a Leitz DMRBE microscope using a Leica DFC290 camera computer-controlled by Leica Q with Standard v. 3.5.0 software (Leica Microsystems, Wetzlar, Germany). A photograph of the same size was taken of a graticule to measure the photographic magnification. To calculate the number of green algae in each photo, every image was opened in ImageJ software (version 1.51j8, National Institute of Health, Bethesda, MD, USA, https://imagej.nih.gov/ij/) and converted to 8-bit grey-scales. The photos were then inverted and threshold defined in order to select the individual algae. The algae were counted by analyzing the particles of each image using the particle analysis tool. The mean number of algae from the 10 images was then calculated, and using the measurements from the graticule, the mean number of algae on the whole cover slip could be calculated as the number of algae in 4 µL. This was then used to finally work out the number of algae per mL.

ASTM medium and Scottish marine algae in each vial were exchanged half way through the 4-day incubation for fresh medium. Neonates were removed within 24 h, and animals that had died before the end of the experiment were dissected as soon as was possible. The swimming strength (as estimated by looking at swimming speed by eye), number of eggs in the brood chamber, egg development time, number of neonates released, and number of completely molted carapaces shed was recorded daily by examining each individual experimental animal.

### 4.3. Dissection of Optic Ganglia and Brains

After the experiment, the animals were transferred to Sylgard^®^–coated Falcon^®^ Petri dishes (Silicone elastomer 184: Dow Corning Co, Midland, MI, USA; dishes: no. 351008, Becton Dickinson Co, Franklin Lakes, NJ, USA) containing *Daphnia* saline (3.565 g L^−1^ NaCl, 0.224 g L^−1^ KCL, 0.203 g L^−1^ MgCl_2_ × 6H_2_O, 1.668 g L^−1^ HEPES, Waters MilliQ^®^-filtered H_2_O, pH adjusted to 7.3 with 0.1 M NaOH) [58]. With the use of microdissection scissors and fine forceps, both second antennae were removed at the base close to the carapace, and the headshield was opened by cutting the dorsal carapace from the anterior end of the heart to the anterior side of the hepatic caeca. The foregut and hepatic caeca were carefully removed creating a flow-through area for the later applied fixative. A dorso-ventral cut posterior to the mandibles was made, allowing the head to be removed from the rest of the body. These heads were then bathed in Zamboni fixative consisting of 4% paraformaldehyde and 15% picric acid in 0.1 M sodium phosphate buffer at pH 7.3 [59] (Chemicals from Sigma-Aldrich/Merck KG, Darmstadt, Germany) for 1 h at room temperature. After this time, the dissected heads were put back into the *Daphnia* saline. The mandibles were removed and the cuticle was carefully torn apart, breaking the connection between the eye and cuticle. The complex of the optic ganglia and the central brain together with the compound eye were then further fixed in Zamboni fixative once more and left overnight at 4 °C for tissue stabilization.

### 4.4. Immunohistochemistry and Confocal Microscopy

For immunohistochemical labelling, preparations were first washed for 4 × 15 min with 0.1 M phosphate buffered saline (PBS) on a Gyrotory^®^ shaker (New Brunswick Scientific G2, Edison, NJ, USA) to remove the fixative. Preparations were then incubated at room temperature in humid conditions for 24 h in rabbit anti-tyrosine hydroxylase (TH) primary antibody (Sino Biological Inc., Beijing, China; no. 50997) diluted 1:2000 in Tris-hydroxyl-amino methane-buffer at pH 7.4 containing 0.3 M NaCl and 0.5% Triton-X-100 detergent (TBTX0.5) with 0.02% sodium azide. This was followed by 4 × 15 min washes in Tris-hydroxyl-amino methane-buffer containing 0.3 M NaCl and 0.1% Triton-X-100 detergent (TBTX0.1) and then the preparations were incubated at room temperature for 1 h 45 min in darkness in goat-anti-rabbit-IgG-FITC conjugate secondary antibody (F0382, Sigma-Aldrich/Merck KG, Darmstadt, Germany) diluted 1:100 in TBTX0.5 with 0.02% sodium azide. After 3 × 15 min washes in TXTB0.1 and 1 × 15 min wash in PBS, preparations were mounted in 80% aqueous glycerol containing 50 mg mL^−1^ 1,4-diazabicyclo (2.2.2) octane (Fluka, Sigma-Aldrich/Merck KG, Darmstadt, Germany) as anti-fading agent.

To obtain a more detailed overview of the dopaminergic system of *D. magna*, preparations for confocal microscopy were triple-labelled by in addition using antibodies revealing entire ganglionic neuropile areas via labelling synapsin, a protein found in all synaptic contacts [60,61]. Together with the TH antibody, a mouse monoclonal anti-synapsin primary antibody (DSHB, Developmental Studies Hybridoma Bank, University of Iowa, Iowa, IA, USA, code 3C11 = anti SYNORF1, [60]) diluted 1:100 in TBTX0.5 with 0.02% sodium azide was applied. After several washes in TBTX0.1, a goat anti-rabbit-IgG-FITC conjugate secondary antibody (Sigma F0382) diluted 1:100 in TBTX0.5 with 0.02% sodium azide was added as well as a goat anti-mouse-IgG (H + L)-Alexa Fluor 546 conjugated secondary antibody (Life Technologies 11003, Thermo Fisher Scientific, Waltham, MA. USA) diluted 1:500 in TBTX0.5 with 0.02% sodium azide for 1 h at room temperature to reveal both TH and synapsin. Here, finally a DAPI stain (4′,6-Diamidine-2′-phenylindole dihydrochloride, Sigma-Aldrich/Merck KG, Darmstadt, Germany) diluted to 1 µg mL^−1^ in TBTX0.5 with 0.02% sodium azide was added to stain the DNA in cell nuclei. This triple staining was used to provide background staining (for neuropiles and cell nuclei) to show where the dopaminergic neurons are in relation to the whole optic ganglia and brain. The preparations were analyzed and imaged using a Zeiss LSM 780 confocal laser-scanning microscope (Carl Zeiss, Oberkochen, Germany) operating with lasers at 405 nm (for DAPI), 488 nm (for FITC), 561 nm (for Alexa Fluor 546) wavelengths and Zeiss Zen 2012 LSM Confocal software (Carl Zeiss, Oberkochen, Germany). Final pictures were edited and slightly adjusted for contrast and brightness using Corel Photopaint v. X7 and assembled in CorelDraw v. X7 (Corel Co, Ottawa, ON, Canada).

### 4.5. Imaging and Morphometry for Toxicity Test Evaluation

Immunofluorescence imaging and quantification were performed as previously described [49]. Preparations were imaged with a Zeiss Axioscope-2 microscope (Carl Zeiss, Oberkochen, Germany) with a Lambda 10-c filter wheel, a motorized focus, equipped with epifluorescence (FITC filter system—excitation filter band pass 485 ± 20 nm, emission filter band pass 515–565 nm) computer-controlled by Zeiss Axiovision v. 4.8.2.0 software (Carl Zeiss, Oberkochen, Germany) allowing for merging and smoothing of several Z-series focus planes (extended focus module). A valid comparability of measurements of the different preparations was ensured by taking all photographs at a fixed exposure time of 900 ms and by using identical microscope settings of a 40× objective and 1.6× secondary Zeiss Optovar magnification [48] with an optical thickness of 1 µm by an AxioCam HR camera (Carl Zeiss, Oberkochen, Germany) set to 1388 × 1040 pixels standard mono using Z-stacking. All Z-stack images were merged into an extended focus high projection single image.

### 4.6. Data Analysis

Fluorescence intensity for the toxicity test was quantified using ImageJ software by analyzing Average Pixel Intensity (API) [62], which was calculated manually. Therefore, each of the images were introduced into ImageJ two times; these were then converted to 8-bit grey-scale images of 8 bit. One of the images was then inverted and threshold defined for the selection of the relative fluorescent neurons. To have comparable measurements of each preparation, the same optimum threshold level was used for each image. The threshold-defined selection from the first image was then transferred to the second image. For the calculation of background API, we chose the part outside of the selection and measured its average pixel intensity. This background pixel intensity was thereafter subtracted from every pixel in the image to remove it. The selected fluorescent neuronal areas were again superimposed to the image from which the background had been subtracted. In order to measure only specific fluorescence, every image was background-subtracted so that from every threshold-defined region its API and fluorescent area could be calculated. Instead of API, the Integrated Density ((ID), which equals fluorescent area × average pixel intensity) was, however, used as a valid representation of the fluorescence of the neurons. This is because ID considers the labelled area as well as API [63]. Thereby, ID is a good representation of dopamine immunoreactivity in individual optic ganglia/brain-complexes (OGBC) of the experimental animals; pictures of optic ganglia and brains were usually analyzed separately but derived values were later combined. All statistical tests were performed with PAST (http://palaeo-electronica.org/2001_1/past/issue1_01.htm) [64].

## References

[1] Kurland L.T., Mulder D.W. (1954). Epidemiologic investigations of amyotrophic lateral sclerosis. I. Preliminary report on geographic distribution, with special reference to the Mariana Islands, including clinical and pathologic observations. Neurology.

[2] Cox P.A., Banack S.A., Murch S.J. (2003). Biomagnification of cyanobacterial neurotoxins and neurodegenerative disease among the Chamorro people of Guam. Proc. Natl. Acad. Sci. USA.

[3] Holtcamp W. (2012). The emerging science of BMAA: Do cyanobacteria contribute to neurodegenerative disease?. Environ. Health Perspect..

[4] Cox P.A., Sacks O.W. (2002). Cycad neurotoxins, consumption of flying foxes, and ALS-PDC disease in Guam. Neurology.

[5] Banack S.A., Cox P.A. (2003). Biomagnification of cycad neurotoxins in flying foxes: Implications for ALS-PDC in Guam. Neurology.

[6] Rao S.D., Banack S.A., Cox P.A., Weiss J.H. (2006). BMAA selectively injures motor neurons via AMPA/kainate receptor activation. Exp. Neurol..

[7] Lobner D., Piana P.M.T., Salous A.K., Peoples R.W. (2007). β-*N*-methylamino-l-alanine enhances neurotoxicity through multiple mechanisms. Neurobiol. Dis..

[8] Liu X., Rush T., Zapata J., Lobner D. (2009). β-*N*-methylamino-l-alanine induces oxidative stress and glutamate release through action on system Xc(-). Exp. Neurol..

[9] Murch S.J., Cox P.A., Banack S.A., Steele J.C., Sacks O.W. (2004). Occurrence of β-methylamino-l-alanine (BMAA) in ALS/PDC patients from Guam. Acta Neurol. Scand..

[10] Pablo J., Banack S.A., Cox P.A., Johnson T.E., Papapetropoulos S., Bradley W.G., Buck A., Mash D.C. (2009). Cyanobacterial neurotoxin BMAA in ALS and Alzheimer’s disease. Acta Neurol. Scand..

[11] Mello F.D., Braidy N., Marcal H., Guillemin G., Nabavi S.M., Neilan B.A. (2018). Mechanisms and effects posed by neurotoxic products of cyanobacteria/microbial eukaryotes/dinoflagellates in algae blooms: A review. Neurotox. Res..

[12] Bienfang P.K., Defelice S.V., Laws E.A., Brand L.E., Bidigare R.R., Christensen S., Trapido-Rosenthal H., Hemscheidt T.K., McGillicuddy D.J., Anderson D.M. (2011). Prominent human health impacts from several marine microbes: History, ecology, and public health implications. Int. J. Microbiol..

[13] Cox P.A., Banack S.A., Murch S.J., Rasmussen U., Tien G., Bidigare R.R., Metcalf J.S., Morrison L.F., Codd G.A., Bergman B. (2005). Diverse taxa of cyanobacteria produce β-*N*-methylamino-l-alanine, a neurotoxic amino acid. Proc. Natl. Acad. Sci. USA.

[14] Jiang L., Eriksson J., Lage S., Jonasson S., Shams S., Mehine M., Ilag L.L., Rasmussen U. (2014). Diatoms: A novel source for the neurotoxin BMAA in aquatic environments. PLoS ONE.

[15] Lage S., Costa P.R., Moita T., Eriksson J., Rasmussen U., Rydberg S.J. (2014). BMAA in shellfish from two Portuguese transitional water bodies suggests the marine dinoflagellate *Gymnodinium catenatum* as a potential BMAA source. Aquat.Toxicol..

[16] Metcalf J.S., Banack S.A., Lindsay J., Morrison L.F., Cox P.A., Codd G.A. (2008). Co-occurrence of β-*N*-methylamino-l-alanine, a neurotoxic amino acid with other cyanobacterial toxins in British waterbodies, 1990–2004. Environ. Microbiol..

[17] Esterhuizen M., Downing T.G. (2008). β-*N*-methylamino-l-alanine (BMAA) in novel South African cyanobacterial isolates. Ecotoxicol. Environ. Saf..

[18] Faassen E.J., Gillissen F., Zweers H.A., Lurling M. (2009). Determination of the neurotoxins BMAA (β-*N*-methylamino-l-alanine) and DAB (alpha-,gamma-diaminobutyric acid) by LC-MSMS in Dutch urban waters with cyanobacterial blooms. Amyotroph. Lateral Scler..

[19] Horner R.D., Kamins K.G., Feussner J.R., Grambow S.C., Hoff-Lindquist J., Harati Y., Mitsumoto H., Pascuzzi R., Spencer P.S., Tim R. (2003). Occurrence of amyotrophic lateral sclerosis among Gulf War veterans. Neurology.

[20] Cox P.A., Richer R., Metcalf J.S., Banack S.A., Codd G.A., Bradley W.G. (2009). Cyanobacteria and BMAA exposure from desert dust: A possible link to sporadic ALS among Gulf War veterans. Amyotroph. Lateral Scler..

[21] Craighead D., Metcalf J.S., Banack S.A., Amgalan L., Reynolds H.V., Batmunkh M. (2009). Presence of the neurotoxic amino acids β-*N*-methylamino-l-alanine (BMAA) and 2,4-diamino-butyric acid (DAB) in shallow springs from the Gobi Desert. Amyotroph. Lateral Scler..

[22] Li A., Tian Z., Li J., Yu R., Banack S.A., Wang Z. (2010). Detection of the neurotoxin BMAA within cyanobacteria isolated from freshwater in China. Toxicon.

[23] Hilborn E.D., Beasley V.R. (2015). One health and cyanobacteria in freshwater systems: Animal illnesses and deaths are sentinel events for human health risks. Toxins.

[24] Spencer P., Ohta M., Palmer V. (1987). Cycad use and motor neurone disease in Kii peninsula of Japan. Lancet.

[25] Cox P.A., Davis D.A., Mash D.C., Metcalf J.S., Banack S.A. (2016). Dietary exposure to an environmental toxin triggers neurofibrillary tangles and amyloid deposits in the brain. Proc. R. Soc. B: Biol. Sci..

[26] Caller T., Henegan P., Stommel E. (2018). The Potential Role of BMAA in Neurodegeneration. Neurotox. Res..

[27] Mimuro M., Yoshida M., Kuzuhara S., Kokubo Y. (2018). Amyotrophic lateral sclerosis and parkinsonism-dementia complex of the Hohara focus of the Kii Peninsula: A multiple proteinopathy?. Neuropathology.

[28] Al-Chalabi A., Hardiman O. (2013). The epidemiology of ALS: A conspiracy of genes, environment and time. Nat. Rev. Neurol..

[29] Al-Chalabi A., Calvo A., Chio A., Colville S., Ellis C.M., Hardiman O., Heverin M., Howard R.S., Huisman M.H.B., Keren N. (2014). Analysis of amyotrophic lateral sclerosis as a multistep process: A population-based modelling study. Lancet Neurol..

[30] Degerholm J., Gundersen K., Bergman B., Soderback E. (2006). Phosphorus-limited growth dynamics in two Baltic Sea cyanobacteria, *Nodularia* sp. and *Aphanizomenon* sp.. FEMS Microbiol. Ecol..

[31] Jonasson S., Eriksson J., Berntzon L., Spacil Z., Ilag L.L., Ronnevi L.O., Rasmussen U., Bergman B. (2010). Transfer of a cyanobacterial neurotoxin within a temperate aquatic ecosystem suggests pathways for human exposure. Proc. Natl. Acad. Sci. USA.

[32] Jiang L., Kiselova N., Rosén J., Ilag L.L. (2014). Quantification of neurotoxin BMAA (β-*N*-methylamino-l-alanine) in seafood from Swedish markets. Sci. Rep..

[33] Salomonsson M.L., Fredriksson E., Alfjorden A., Hedeland M., Bondesson U. (2015). Seafood sold in Sweden contains BMAA: A study of free and total concentrations with UHPLC–MS/MS and dansyl chloride derivatization. Toxicol. Rep..

[34] Lage S., Annadotter H., Rasmussen U., Rydberg S. (2015). Biotransfer of β-*N*-methylamino-l-alanine (BMAA) in a eutrophicated freshwater lake. Mar. Drugs.

[35] Esterhuizen-Londt M., Wiegand C., Downing T.G. (2015). β-*N*-methylamino-l-alanine (BMAA) uptake by the animal model, *Daphnia magna* and subsequent oxidative stress. Toxicon.

[36] Lürling M. (2003). Effects of microcystin-free and microcystin-containing strains of the cyanobacterium *Microcystis aeruginosa* on growth of the grazer *Daphnia magna*. Environ. Toxicol..

[37] McQueen D.J., Post J.R., Mills E.L. (1986). Trophic Relationships in Freshwater Pelagic Ecosystems. Can. J. Fish. Aquat. Sci..

[38] Martins J., Oliva Teles L., Vasconcelos V. (2007). Assays with *Daphnia magna* and *Danio rerio* as alert systems in aquatic toxicology. Environ. Int..

[39] Lürling M., Faassen E.J., Van Eenennaam J.S. (2011). Effects of the cyanobacterial neurotoxin β-*N*-methylamino-l-alanine (BMAA) on the survival, mobility and reproduction of *Daphnia magna*. J. Plankton Res..

[40] Kokubo Y., Ishii K., Morimoto S., Mimuro M., Sasak R., Murayama S., Kuzuhara S. (2017). Dopaminergic positron emission tomography study on Amyotrophic lateral sclerosis/Parkinsonism–Dementia complex in Kii, Japan. J. Alzheimers Dis. Parkinsonism.

[41] Takahashi H., Snow B., Bhatt M.H., Peppard R., Eisen A., Calne D.B. (1993). Evidence for a dopaminergic deficit in sporadic amyotrophic lateral sclerosis on positron emission scanning. Lancet.

[42] Aramant R., Elofsson R. (1976). Distribution of monoaminergic neurons in the nervous system of non-malacostracan crustaceans. Cell Tissue Res..

[43] Ehrenström F., Berglind R. (1988). Determination of biogenic amines in the water flea, *Daphnia magna* (Cladocera, Crustacea) and their diurnal variations using ion-pair reversed phase HPLC with electrochemical detection. Comp. Biochem. Physiol. C Comp. Pharmacol. Toxicol..

[44] Barrozo E.R., Fowler D.A., Beckman M.L. (2015). Exposure to D2-like dopamine receptor agonists inhibits swimming in *Daphnia magna*. Pharmacol. Biochem. Behav..

[45] Weiss L.C., Leese F., Laforsch C., Tollrian R. (2015). Dopamine is a key regulator in the signalling pathway underlying predator-induced defences in *Daphnia*. Proc. R. Soc. B Biol. Sci..

[46] Kress T., Harzsch S., Dircksen H. (2016). Neuroanatomy of the optic ganglia and central brain of the water flea *Daphnia magna* (Crustacea, Cladocera). Cell Tissue Res..

[47] Zhang Q., Keller R., Dircksen H. (1997). Crustacean hyperglycaemic hormone in the nervous system of the primitive crustacean species *Daphnia magna* and *Artemia salina* (Crustacea: Branchiopoda). Cell Tissue Res..

[48] Strauß J., Zhang Q., Verleyen P., Huybrechts J., Neupert S., Predel R., Pauwels K., Dircksen H. (2011). Pigment-dispersing hormone in *Daphnia* interneurons, one type homologous to insect clock neurons displaying circadian rhythmicity. Cell. Mol. Life Sci..

[49] Campos B., Rivetti C., Kress T., Barata C., Dircksen H. (2016). Depressing antidepressant: Fluoxetine affects serotonin neurons causing adverse reproductive responses in *Daphnia magna*. Environ. Sci. Technol..

[50] Purdie E.L., Metcalf J.S., Kashmiri S., Codd G.A. (2009). Toxicity of the cyanobacterial neurotoxin β-*N*-methylamino-l-alanine to three aquatic animal species. Amyotroph. Lateral Scler..

[51] Bernheimer H., Birkmayer W., Hornykiewicz O., Jellinger K., Seitelberger F. (1973). Brain dopamine and the syndromes of Parkinson and Huntington. Clinical, morphological and neurochemical correlations. J. Neurol. Sci..

[52] Jana S., Sinha M., Chanda D., Roy T., Banerjee K., Munshi S., Patro B.S., Chakrabarti S. (2011). Mitochondrial dysfunction mediated by quinone oxidation products of dopamine: Implications in dopamine cytotoxicity and pathogenesis of Parkinson’s disease. Biochim. Biophys. Acta.

[53] Spencer P.S. (1987). Guam ALS/parkinsonism-dementia: A long-latency neurotoxic disorder caused by “slow toxin(s)” in food?. Can. J. Neurol. Sci..

[54] Crocker A.D. (1997). The regulation of motor control: An evaluation of the role of dopamine receptors in the substantia nigra. Rev. Neurosci..

[55] Pistacchi M., Gioulis M., Sanson F., De Giovannini E., Filippi G., Rossetto F., Zambito Marsala S. (2017). Gait analysis and clinical correlations in early Parkinson’s disease. Funct. Neurol..

[56] Paerl H.W., Huisman J. (2008). Climate. Blooms like it hot. Science.

[57] Baird D.J., Soares A.M.V.M., Girling A.E., Barber I., Bradley M.C., Calow P., Lokke H., Tyle H., Bro-Rasmussen F. (1989). The long-term maintenance of *Daphnia magna* for use in ecotoxicity tests: Problems and prospects. Proceedings of the First European Conference on Ecotoxicology.

[58] Van Harreveld A. (1936). A physiological solution for freshwater crustaceans. Proc. Soc. Exp. Biol. Med..

[59] Stefanini M., De Martino C., Zamboni L. (1967). Fixation of ejaculated spermatozoa for electron microscopy. Nature.

[60] Klagges B.R.E., Heimbeck G., Godenschwege T.A., Hofbauer A., Pflugfelder G.O., Reifegerste R., Reisch D., Schaupp M., Buchner S., Buchner E. (1996). Invertebrate synapsins: A single gene codes for several isoforms in *Drosophila*. J. Neurosci..

[61] Harzsch S., Glötzner J. (2002). An immunohistochemical study of structure and development of the nervous system in the brine shrimp *Artemia salina* Linnaeus, 1758 (Branchiopoda, Anostraca) with remarks on the evolution of the arthropod brain. Arthropod Struct. Dev..

[62] Raldúa D., Babin P.J. (2009). Simple, rapid zebrafish larva bioassay for assessing the potential of chemical pollutants and drugs to disrupt thyroid gland function. Environ. Sci. Technol..

[63] Thienpont B., Tingaud-Sequeira A., Prats E., Barata C., Babin P.J., Raldúa D. (2011). Zebrafish eleutheroembryos provide a suitable vertebrate model for screening chemicals that impair thyroid hormone synthesis. Environ. Sci. Technol..

[64] Hammer Ø., Harper D.A.T., Ryan P.D. (2001). PAST: Paleontological statistics software package for education and data analysis. Palaeontol. Electron..

